# Perceived barriers of tobacco dependence treatment: a mixed-methods study among primary healthcare physicians in Armenia

**DOI:** 10.1017/S1463423618000828

**Published:** 2018-11-13

**Authors:** Arusyak Harutyunyan, Armine Abrahamyan, Varduhi Hayrumyan, Varduhi Petrosyan

**Affiliations:** Gerald and Patricia Turpanjian School of Public Health, American University of Armenia, Yerevan, Armenia

**Keywords:** Armenia, barriers, mixed-methods study, primary healthcare physicians, smoking cessation

## Abstract

**Background:**

Despite compelling evidence that physicians play a prominent role in smoking cessation, most smokers do not receive the recommended smoking cessation counseling.

**Aim:**

To identify perceived barriers that hinder primary healthcare physicians (PHPs) from providing smoking cessation treatment to patients in Armenia.

**Methods:**

A sequential exploratory mixed-methods study was conducted among PHPs from two Armenian cities (Yerevan and Gyumri). We implemented qualitative phase through focus group discussions (FGDs) using a semi-structured guide. For the subsequent quantitative phase, the data were collected through cross-sectional survey. A directed deductive content analysis technique was used to analyze the FGDs and questionnaires were analyzed descriptively. Following the data collection (March 2015–May 2016) and descriptive analysis, the qualitative and quantitative data sets were merged by drawing quantitative data onto qualitative categories.

**Findings:**

Overall, 23 PHPs participated in five FGDs and 108 participants completed the survey. Three main categories of barriers were identified: physician-based, patient-based, and system-based barriers. The main physicians-based barriers were insufficient knowledge and inadequate training on tobacco-dependence treatment. Lack of patients’ motivation to quit, poor compliance with the treatment, patients’ withdrawal symptoms were identified as patient-based disincentives. System-based barriers included lack of reimbursement for providing smoking cessation counseling, high price and low availability of smoking cessation medications. Most of the qualitative descriptions were confirmed by quantitative findings.

**Conclusions:**

Targeted interventions are needed to address barriers that limited PHPs’ involvement in providing smoking cessation services in Armenia. There is an urgent need to enhance PHPs’ knowledge and skills in delivering smoking cessation counseling, to increase patients’ demand for smoking cessation services, and to ensure availability and affordability of smoking cessation services in Armenia.

## Introduction

The tobacco epidemic is one of the biggest public health threats killing around 7 million people per year worldwide (World Health Organization, 2018). The number of annual deaths caused by tobacco use has been estimated to increase to eight million by 2030 (World Health Organization, 2017). Smoking is a chronic disease and repeated, opportunity-based interventions initiated by healthcare providers have been shown to be the most effective in addressing physical dependence and modifying deeply ingrained patterns of beliefs and behavior (McIvor *et al*., 2009). Cessation interventions have a mid-term impact on the number of deaths and therefore must be encouraged. If smoking initiation is reduced by 50% by 2020, the number of deaths from tobacco could decrease from 520 million to around 500 million in 2050. Alternatively, if half of the current smokers quit by 2020, the number of deaths from smoking could be reduced from 520 to 340 million in 2050 (World Bank, 1999). The World Health Organization (WHO) Framework Convention on Tobacco Control (FCTC) Article 14 requires parties to take effective measures to promote smoking cessation and appropriate tobacco-dependence treatment. Smoking cessation services and assistance are not available in about 45% of low-income countries (World Health Organization, 2017). Despite compelling evidence that physicians play a prominent role in smoking cessation (Anczak and Nogler, 2003), most smokers do not receive the recommended smoking cessation counseling (Caplan *et al*., 2011). The major obstacles to achieving consistent tobacco dependence treatment include inadequate training (Abdullah and Husten, 2004; Jradi, 2015), lack of knowledge of smoking cessation pharmacotherapy (Young and Ward, 2001), lack of time (Abdullah *et al*., 2006; Blumenthal, 2007), heavy workload (Brotonsc *et al*., 2005), lack of incentives for providing smoking cessation services (Young and Ward, 2001; Brotonsc *et al*., 2005), and low interest among patients (Abdullah *et al*., 2006; Joshi *et al*., 2010). Tobacco smoking is the most prevalent and dangerous behavioral risk factor among the Armenian male population. From 2010 to 2015–2016, the prevalence of daily tobacco smoking in the population 15–49 years old did not change dramatically in Armenia: it decreased from 63.0% to 61.4% for men and from 1.6% to 1.2% for women (Armenia Demographic and Health Survey, 2010; Armenia Demographic and Health Survey, 2015). Despite the small decline in the prevalence, Armenia has the highest male smoking rate in the European region (Armenia Demographic and Health Survey, 2015).

Smoking is also remarkably prevalent among Armenian physicians (48.5% of males, 12.8% of females) and medical students (50.0% of males, 7.7% of females) (Perrin *et al*., 2006). In 2015, primary healthcare (PHC) services in Armenia were delivered through 363 public and 141 private/other PHC units (Petrosyan *et al*., 2017). Public PHC units include urban polyclinics, health centers, and rural ambulatory facilities that work with 552 small centers (called FAPs [Feldsher Acousher Posts]) run by nurses or midwives who are supervised by physicians from a nearby larger PHC unit and legally belong to that unit. PHC services are fully publicly funded for all Armenian citizens (Petrosyan *et al*., 2017). Armenia was the first former Soviet Union country to accede to the WHO/FCTC and soon after, Armenia adopted a national tobacco control law to ban smoking in healthcare, education, and cultural facilities, as well as public transportation (Framework Convention on Tobacco Control, 2011). One of the areas where Armenia’s progress is less than satisfactory is the implementation of the FCTC Article 14. For instance, the tobacco dependence treatment guidelines are not properly implemented in the medical practice and smoking cessation training is not incorporated into the graduate and postgraduate training curricula for health professionals.

The aim of this study was to identify perceived barriers which hinder primary healthcare physicians (PHP) from providing smoking cessation counseling and treatment to patients in Armenia.

## Methods

A sequential exploratory mixed-methods study was conducted among PHPs from two Armenian cities, Yerevan, the capital of Armenia, and Gyumri, the second largest city, located in Shirak region (marz). This is one of the few studies exploring in detail barriers in provision of smoking cessation services within routine care in Armenia. The mixed-methods study design was chosen because of its potential to generate more complete and higher quality data in an area that has not been sufficiently studied (Hadi *et al*., 2013).

### Data collection

For the qualitative phase of the study, the research team developed and implemented focus group discussions (FGDs) with PHPs (general practitioners including family physicians) to clarify their knowledge, attitude, and practices (KAP) regarding smoking cessation, as well as perceived barriers for the provision of smoking cessation services. The FGD participants were identified using a purposive sampling method to obtain pertinent information for the assessment. A semi-structured guide was developed based on the main research questions in order to moderate the FGDs (Box 1). The data collection was conducted in March 2015. The sessions were audio recorded with the permission of all study participants. The mean duration of the FGDs was 38 minutes. The qualitative study followed the research methods of heterogeneity and triangulation, and was terminated when saturation was achieved. After data collection, the research team transcribed the data and analyzed FGD transcripts using directed deductive content analysis techniques (Hsieh and Shannon, 2005). For the subsequent quantitative phase, the data were collected through a cross-sectional survey to evaluate PHPs’ KAP regarding smoking cessation, as well as their confidence and potential barriers in providing smoking cessation counseling. The data collection for quantitative phase was conducted in May 2016. The perceived barriers were measured by providing a list of potential barriers for participants to rate using a three-point scale (‘not a barrier’, ‘somewhat a barrier’, ‘important barrier’). The survey participants were identified through the existing network of family physicians’ association with assistance from Yerevan and Gyumri Municipalities’ Health Departments.

### Data analysis

A single data entry was performed using SPSS 22.0 statistical package followed by logical and range checks to ensure the accuracy of data. Statistical analysis was completed using SPSS 22.0 and STATA 13.0 statistical software. The study team used descriptive statistics to summarize the participants’ characteristics and barriers’ ratings. Following the data collection and descriptive analysis, the qualitative and quantitative data sets were merged by drawing quantitative data onto qualitative categories (Hadi *et al*., 2013). The main categories were grouped into three domains: physician-based barriers, patient-based barriers, and system-based barriers. These three domains served as an initial framework to identify potential barriers in providing smoking cessation services in Armenia. The American University of Armenia Institutional Review Board reviewed and approved the study protocols. All participants gave oral consent before participating in the study.

## Results

### Socio-demographic characteristics of participants

Overall, 23 PHPs (Yerevan (*n* = 12) and Gyumri (*n* = 11)) participated in five FGDs and 108 participants completed the survey (Yerevan (*n* = 75) and Gyumri (*n* = 33)). The mean age of FGD and survey participants was 52.7 (*SD* = 9.8) and 53.2 (*SD* = 10.2), respectively. The great majority of FGD and survey participants were women (100% and 97.2%, respectively) (Table 1).

**Table 1:** Sociodemographic characteristics of the participants

Variables	Survey (*n* = 108) % (*n*)	FGDs (*n* = 23) % (*n*)
Age (years), *M* (*SD*)	53.19 (10.19)	52.70 (9.78)
Gender, female	97.22 (105)	100 (23)
Working years as healthcare physician, *M* (*SD*)	25.41 (12.80)	27.22 (10.08)
Teaching at any educational institution		
Yes	10.19 (11)	–^a^
No	87.96 (95)	–

FGD, focus group discussions.

a The data were not collected among FGDs’ participants.

### Physician-based barriers

Armenian PHPs qualitatively distinguished between their roles in advising patients to quit smoking and actually providing smoking cessation assistance. While these physicians see themselves as providers of preventive healthcare, they did not consider provision of smoking cessation assistance as their responsibility. One of the physicians stated: *‘The polyclinic [primary healthcare facility] is a preventive clinic. It is not a treatment clinic*’. *[PHP, Yerevan]*

Typically, physicians ceased their involvement in provision of smoking cessation services once they provided advice. They did not offer pharmacotherapy or other interventions, such as cognitive behavior therapy or similar interventions. A physician noted: *‘We have never prescribed drugs [for smoking cessation], it is not our duty’. [PHP, Gyumri]*

Importantly, some of the physicians complained that their role as a treating doctor was underestimated and said: *‘Apparently, we are dealing only with prevention; we do not play any role in the treatment of patients. It is very offensive; the physician in the hospital received the same diploma as I did. So why are you underestimating us?’ [PHP, Yerevan]* Some of the physicians believed that pharmacotherapy is not the first method to quit and patients should be referred to other specialist for help: *‘…all over the world people visit psychologists for quitting smoking. Pharmacotherapy is not the primary method thus psychological assistance comes first’ [PHP, Yerevan].* Meanwhile, some of the respondents expressed contradictory opinion regarding feasibility of referring smokers to other specialists: *‘Maybe people visit psychologists for quitting, but in Armenia who visits psychologists?’ [PHP, Yerevan]* Additionally, the Armenian physicians reported that they were reluctant to provide smoking cessation counseling as they were afraid of harming the physician-patient relationship. PHPs’ believed that asking patients about their smoking status could engender confrontation and be ‘offensive’ and ‘harmful’. A PHP said: *‘Advising a person not to smoke may lead to conflict situation.’ [PHP, Yerevan]* Some of the Armenian PHPs considered smoking as a culturally sensitive issue, particularly among women, and preferred asking about the smoking status of men rather than women, and asking their relatives rather than their patients. One of the physicians noted: *‘It is a very sensitive issue. You should not impose, but you should explain. If the person is your relative, you can try to convince him/her to quit; otherwise, they may think that you are very talkative’. [PHP, Yerevan]* Most of the physicians from Gyumri reported that they ask the smoking status of only their male patients. The majority of PHPs did not have formal training on smoking cessation interventions and were lacking appropriate knowledge: *‘During the training courses they [organizers] only mentioned about smoking as a risk factor, but did not mention about other aspects of smoking cessation’. [PHP, Yerevan]* The majority of participants were skeptical about their ability to prescribe pharmacotherapy and felt more competent to offer behavioral counseling to their smoking patients: *‘We are not knowledgeable enough [to prescribe smoking cessation drugs], we are not professionals in it’. [PHP, Yerevan]* In fact, some PHPs had developed some smoking cessation methods based on their own beliefs: *‘I do not advise them to quit, I suggest minimizing it [the number of cigarettes]. I suggest that as soon as you reach the middle of the cigarette, throw it away, as at the end it contains much more nicotine’. [PHP, Yerevan].*

Some of the physician-based qualitative descriptions were confirmed by quantitative findings. A high proportion of respondents reported having insufficient training and knowledge on smoking cessation interventions as important barriers that hinder them from helping patients to quit smoking 45.4% and 42.0%, respectively (Table 2).

**Table 2:** Perceived barriers in providing tobacco dependence treatment, *n* = 108

How would you rate the following as barriers that hinder you from helping patients to stop smoking?	Total % (*n*)
**Physician-based barriers**	
Insufficient knowledge on smoking cessation interventions	
Not a barrier	11.11 (12)
Somewhat a barrier	46.30 (50)
Important barrier	41.67 (42)
No formal training on smoking cessation interventions	
Not a barrier	15.74 (17)
Somewhat a barrier	38.89 (42)
Important barrier	45.37 (49)
**Patient-based barriers**	
Patients have more immediate health problems to be addressed	
Not a barrier	42.59 (46)
Somewhat a barrier	38.89 (42)
Important barrier	17.59 (19)
Patients are not interested in receiving smoking cessation information	
Not a barrier	28.70 (31)
Somewhat a barrier	46.30 (50)
Important barrier	25.00 (27)
Patients do not comply with information given on smoking cessation	
Not a barrier	9.26 (10)
Somewhat a barrier	44.44 (48)
Important barrier	46.30 (50)
**System-based barriers**	
Lack of time/time with patients is limited	
Not a barrier	19.44 (21)
Somewhat a barrier	50.00 (54)
Important barrier	30.56 (33)
Lack of smoking cessation specialists to refer patients to for further assistance	
Not a barrier	18.52 (20)
Somewhat a barrier	37.04 (40)
Important barrier	43.52 (47)
Lack of patient education material (brochures/pamphlets)	
Not a barrier	9.26 (10)
Somewhat a barrier	46.30 (50)
Important barrier	43.52 (47)
Lack of smoking cessation guidelines	
Not a barrier	12.96 (14)
Somewhat a barrier	47.22 (51)
Important barrier	38.89 (42)

### Patient-based barriers

PHPs believed that engaging patients with a low level of motivation in cessation activities were a difficult and challenging issue, thus, they emphasized that patients should rely on their willpower for quitting and choose smoking-cessation methods on their own. One of the physicians said: ‘*It does not matter how much women or children ask the father or husband to quit; each person should have willingness to quit smoking’. [PHP, Yerevan]* Importantly, the majority of physicians believed that the main motivators which trigger patients’ desire to quit smoking were the smoking-related financial burden and the development of chronic health conditions (such as myocardial infarction and cancer). One physician noted: *‘…I have patients that quit smoking after stroke and myocardial infarction and do not return to it again. The same person before stroke said that it is impossible for him to quit’. [PHP, Yerevan]*

Physicians also mentioned that even in rare cases when they prescribed smoking cessation medication, the patients did not adhere to the treatment plan, and thus they could not recall any successful cases of patients quitting as a result of pharmacotherapy. *‘You can prescribe, but I do not remember any case when a patient followed my advice and took smoking cessation drugs’. [PHP, Yerevan]* PHPs reported that they often did not pursue smoking cessation among their patients because while trying to stop smoking their patients experienced loss of teeth, weight gain, and nervous tension. One of the physicians noted: *‘…gaining weight, losing teeth, coughing… because of these reasons they [patients] start smoking again’. [PHP, Gyumri]*

Armenian physicians also tended to avoid discussion of smoking cessation with special patient subgroups (elderly patients, patients with other severe comorbidities, patients with other addiction disorders) because of the misinformed belief that smoking ‘already harmed’ them and their health problems take precedence over smoking cessation counseling: *‘I think that there is no need to convince a 70 years old person to quit smoking, as well as a patient with a severe condition. They already have serious problems and stress associated with quitting will harm them even more, rather than the nicotine’. [PHP, Yerevan]*

According to 42.6% (*n* = 46) of survey respondents, patients’ other immediate health problems to be addressed were not barriers for providing smoking cessation. While ranking patients’ interest in receiving smoking cessation information, only 25.0% of physicians perceived patients’ lack of interest as an important barrier and 46.3% (*n* = 50) ranked it as somewhat a barrier (Table 2). Patients’ noncompliance with information concerning smoking cessation was identified as a top-ranked patient-based barrier. Almost half of all participants ranked it as an important or somewhat important barrier 46.3% (*n* = 50) and 44.4% (*n* = 48), respectively.

### System-based barriers

All PHPs reported that through the pay for performance (P4P) system, they receive bonus payments based on their performance with respect to several indicators, including healthy lifestyle counseling among adults. PHPs clarified that healthy lifestyle counseling includes consultation on alcohol and substance abuse, physical activity, dietary habits, as well as provision of smoking cessation services. PHPs also added that it is mandatory to report provision of healthy lifestyle counseling in the medical records of the patients. *‘Smoking is included in counseling on healthy lifestyle, but we do not mention it as a separate point [in medical records]’. [PHP, Yerevan]* The PHPs further elaborated that they are not required to report the smoking status of the patients, since it is not included among the traditional vital signs, and there is no separate place for recording it within the medical records. One of the physicians said: *‘When I was in clinical internship, we recorded about non-healthy behavior (smoking, coffee) of our patients in the medical cards. Now we do not have a special place or a column where we can write this [smoking status of the patient]’. [PHP, Yerevan]*

PHPs complained that unreasonable paperwork and the pressure of an excessive workload were limiting their time spent for interacting with their patients. They felt distracted by numerous obligations and often spoke about being too busy to provide smoking cessation counseling. One physician noted: ‘*Usually I am so busy with paperwork that my patients say that I do not even look at their faces’. [PHP, Yerevan]* Limited availability of smoking cessation products in the Armenian pharmaceutical market was stressed as another obstacle for providing effective smoking cessation services. One of the physicians noted: *‘Sometimes we can prescribe the medication; and then the patient comes back and says that it is almost several months that this drug is still not available in the market. I feel very embarrassed’. [PHP, Yerevan]* Many of the respondents mentioned that because of the high price, their patients did not use smoking cessation medications: *‘It [pharmacotherapy] is very costly. People in developed countries receive a large amount of nicotine gums. In Armenia, smoking cessation drugs are very expensive, as very few companies import them to our market’. [PHP Yerevan]*

The results from the quantitative study revealed that over 80.6% of participants ranked lack of time and lack of smoking cessation specialists for referring patients as important or somewhat important barriers (Table 2). Overall, 89.8% of participants considered lack of patient educational materials as an important or somewhat important barrier and 86.1% of PHPs reported that lack of smoking cessation guidelines was an important or somewhat important barrier in delivering evidence-based smoking cessation interventions (Table 2).

## Discussion

The study results provided an insight into the most common perceived barriers that hinder Armenian PHPs from providing smoking cessation treatment to their patients. The main findings of this study converged upon three overarching categories: physician-based barriers; patient-based barriers; and system-based barriers. The main physicians-based barriers were insufficient knowledge and inadequate training on tobacco dependence treatment. Lack of patients’ motivation to quit, poor compliance with the treatment, patients’ withdrawal symptoms were identified as patient-based disincentives. System-based barriers included lack of reimbursement for providing smoking cessation counseling, high price and low availability of smoking cessation medications.

Inadequate training of PHPs on tobacco dependence and its treatment is one of the major obstacles to acquiring consistent and effective treatment of tobacco dependence (Abdullah and Husten, 2004; Jradi, 2015). Majority of Armenian physicians had never received any formal training or attempted to increase their knowledge of smoking cessation either during preservice or in-service trainings. Participation in smoking cessation trainings (Young and Ward, 2001) doubled the likelihood of offering assistance to smoking patients compared to non-trained physicians (Cummings, 1989; Mejia *et al*., 2016). Study conducted by Caplan *et al*. (Caplan *et al*., 2011) showed that smoking cessation rate among patients can be greatly improved by encouraging physicians’ compliance with the smoking cessation guidelines. The findings of our study indicated that the majority of Armenian PHPs were not aware of the smoking cessation guideline developed by the National Institute of Health. The Ministry of Health of the Republic of Armenia approved ‘The guideline for tobacco cessation counseling and treatment’ for use by PHPs in 2009, but no further steps were undertaken to enable the PHPs to implement the guideline. Our findings are similar to those of other studies in that physicians generally failed to adhere to all of the components of the smoking cessation guidelines (Ferketich *et al*., 2006; Jordan *et al*., 2006). Consistent with the study carried out across 11 European countries, our findings suggest that lack of reimbursement for providing smoking cessation was considered as a major barrier (Young and Ward, 2001; Brotonsc *et al*., 2005). In 2015, results-based financing has been integrated into the Armenian national health care financing system (Petrosyan *et al*., 2017). It covers all primary healthcare facilities in the country and consists of two components: open enrollment and pay for performance (P4P). Through the P4P component, PHPs receive bonus payments based on their performance with respect to 27 indicators (Petrosyan *et al*., 2017). Our participants shared that they were receiving financial benefits for performing activities to change their patients’ health behaviors such as alcohol and substance abuse, physical activity, dietary habits, and smoking, thought they did not receive bonus payments for providing smoking cessation counseling separately. Similar to many other studies, we found that the overwhelming majority of Armenian PHPs considered providing smoking cessation as too time-consuming and that the time spent was not worth the efforts as few patients gave up smoking (Brotonsc *et al*., 2005; Abdullah *et al*., 2006; Blumenthal, 2007). This might be explained with the fact that physicians are overwhelmed with paperwork which restricts the time spent with patients and influences PHPs’ decision to provide smoking cessation services. The findings of our study also revealed that Armenian physicians appeared to prioritize smoking cessation counseling based on patients’ socio-demographic characteristics (eg, age, gender), as well as diagnosis at the time of the visit. They preferred to discuss smoking only with those patients who expressed explicit concern about smoking, as they were afraid of harming physician–patient relationship. Study conducted by Helgason *et al*. stated that substantial number of physicians reported that they felt uncomfortable discussing patients’ smoking behavior (Helgason and Lund, 2002). Smoking was considered as a culturally sensitive issue by some of the PHPs among women and they preferred checking smoking status of men rather than women. Armenian physicians also tend to miss the opportunity to discuss smoking with special patient subgroups (eg, elderly patients, patients with other severe comorbidities) because of the misbelief that smoking ‘already harmed’ them and their health problems take precedence over the smoking cessation counseling. Similarly, study conducted by Coleman *et al.* revealed that smokers with previous psychological morbidity and addictions were encouraged to postpone their quit attempts, as physicians felt that smoking harmed these people less than their addictions (Coleman *et al*., 2000). Armenian PHPs mostly see themselves as providers of preventive services. In spite of acknowledging their role in advising patients to quit, the overwhelming majority of them did not consider provision of smoking cessation assistance as a part of their job. This perceived role was often reflected in physicians’ practice of not offering assistance to their patients which is consistent with the results of a multi-country literature review that covers 29 European countries (Stead *et al*., 2009). The review indicates that although many physicians reported about asking patients’ smoking status and always advising them to quit, the proportion of physicians always offering more intensive interventions or pharmacotherapy for cessation is generally low (Stead *et al*., 2009).

Armenian physicians believed that they face a number of barriers from patients’ side (such as lack of motivation, patients’ poor compliance with the treatment and withdrawal symptoms experienced by patients) that limited their involvement in providing smoking cessation services. The findings are congruent with those in the international literature (Abdullah *et al*., 2006; Blumenthal, 2007; Joshi *et al*., 2010). While discussing about barriers in provision of smoking cessation, both physicians and patient smokers agreed that the key barriers to smoking cessation were craving/physical addiction and smokers’ concern of withdrawal symptoms if they attempted quitting.

### Study strengths and limitations

Our study utilized mixed-method design that gives an advantage to explore the most prevalent barriers to the delivery of smoking cessation services in Armenia. Utilization of mixed-methods study design enabled comparison and integration of qualitative and quantitative data sources to assess the trustworthiness of both the qualitative and quantitative data and subsequent interpretations. The qualitative and quantitative studies were merged following the initial data analysis, thus the study design and analysis were independent from each other. The overwhelming majority of the participants were women which reflects the existing gender composition of general practitioners in Armenia (91.4%) (Health System Performance Assessment, 2016). Our study is limited by being conducted only in two cities among PHPs that were selected by nonrandom sampling. These may affect the generalizability of the findings to other settings in Armenia. In addition, the study evaluated barriers only from physicians’ perspective. Future studies need to explore patients’ perspective on the issue.

## Conclusion

Targeted interventions are needed to address the barriers that limit PHPs’ involvement in providing comprehensive smoking cessation services in Armenia. There is an urgent need to arm PHPs’ with the evidence-based smoking cessation counseling and treatment knowledge and skills through implementation of the existing National Smoking Cessation Guideline into the PHPs’ regular practice, and incorporation of tobacco dependence treatment training into the preservice and in-service training curricula of all health professionals. Performance-based reimbursement mechanisms might be considered as a method for motivating PHPs to provide smoking cessation services. Additional strategies should be explored to increase demand and compliance for smoking cessation services through educational programs and public awareness campaigns. Furthermore, system-wide changes should be considered to strengthen a sustainable infrastructure to ensure availability and affordability of smoking cessation services in Armenia.

Our study findings may have practical implications both locally and regionally, particularly for low- and middle-income countries with slow and/or insufficient implementation of the FCTC Article 14, one of the most under-used tobacco control measures. The explored barriers could enable policy makers to identify the key, effective measures needed to promote tobacco cessation and incorporate tobacco dependence treatment into national tobacco control programs and healthcare system.
